# The Value of MRI-Based Radiomics in Predicting the Pathological Nodal Status of Rectal Cancer: A Systematic Review and Meta-Analysis

**DOI:** 10.3390/bioengineering12070786

**Published:** 2025-07-21

**Authors:** David Luengo Gómez, Marta García Cerezo, David López Cornejo, Ángela Salmerón Ruiz, Encarnación González-Flores, Consolación Melguizo Alonso, Antonio Jesús Láinez Ramos-Bossini, José Prados, Francisco Gabriel Ortega Sánchez

**Affiliations:** 1 Instituto de Investigación Biosanitaria de Granada (ibs.GRANADA), 18012 Granada, Spain; dcornejo@ugr.es (D.L.C.); melguizo@ugr.es (C.M.A.); jcprados@ugr.es (J.P.); 2 Department of Radiology, Hospital Universitario Virgen de las Nieves, 18014 Granada, Spain; david.luengo.sspa@juntadeandalucia.es (D.L.G.); angela.salmeron.sspa@juntadeandalucia.es (Á.S.R.); 3 Center for Genomics and Oncological Research—Centro de Genómica e Investigación Oncológica (GENYO), 18016 Granada, Spain; marta.gcerezo@genyo.es (M.G.C.); gabriel.ortega@genyo.es (F.G.O.S.); 4 Department of Medical Oncology, Hospital Universitario Virgen de las Nieves, 18014 Granada, Spain; encarnacion.gonzalez.flores.sspa@juntadeandalucia.es (E.G.-F.); 5 Institute of Biopathology and Regenerative Medicine (IBIMER), University of Granada, 18100 Granada, Spain; 6 Center of Biomedical Research (CIBM), University of Granada, 18100 Granada, Spain; 7 Department of Human Anatomy and Embryology, School of Medicine, University of Granada, 18016 Granada, Spain

**Keywords:** radiomics, magnetic resonance imaging, lymph node, staging, precision, machine learning

## Abstract

**Background:** MRI-based radiomics has emerged as a promising approach to enhance the non-invasive, presurgical assessment of lymph node staging in rectal cancer (RC). However, its clinical implementation remains limited due to methodological variability in published studies. We conducted a systematic review and meta-analysis to synthesize the diagnostic performance of MRI-based radiomics models for predicting pathological nodal status (pN) in RC. **Methods:** A systematic literature search was conducted in PubMed, Web of Science, and Scopus for studies published until 31 December 2024. Eligible studies applied MRI-based radiomics for pN prediction in RC patients. We excluded other imaging sources and models combining radiomics and other data (e.g., clinical). All models with available outcome metrics were included in data analysis. Data extraction and quality assessment (QUADAS-2) were performed independently by two reviewers. Random-effects meta-analyses including hierarchical summary receiver operating characteristic (HSROC) and restricted maximum likelihood estimator (REML) analyses were conducted to pool sensitivity, specificity, area under the curve (AUC), and diagnostic odds ratios (DORs). Sensitivity analyses and publication bias evaluation were also performed. **Results:** Sixteen studies (*n* = 3157 patients) were included. The HSROC showed pooled sensitivity, specificity, and AUC values of 0.68 (95% CI, 0.63–0.72), 0.73 (95% CI, 0.68–0.78), and 0.70 (95% CI, 0.65–0.75), respectively. The mean pooled AUC and DOR obtained by REML were 0.78 (95% CI, 0.75–0.80) and 6.03 (95% CI, 4.65–7.82). Funnel plot asymmetry and Egger’s test (*p* = 0.025) indicated potential publication bias. **Conclusions:** Overall, MRI-based radiomics models demonstrated moderate accuracy in predicting pN status in RC, with some studies reporting outstanding results. However, heterogeneity in relevant methodological approaches such as the source of MRI sequences or machine learning methods applied along with possible publication bias call for further standardization and preclude their translation to clinical practice.

## 1. Introduction

Colorectal cancer is the third most prevalent cancer worldwide and the second most common cause of oncological death, considering both sexes [1]. In addition, there is a concerning incidence increase in young populations. Rectal cancer represents approximately one third of colorectal neoplasms and has several particularities that translate into a specific biological behavior and clinical management. Fifteen percent of all RC diagnoses in 2020 were estimated to occur in individuals under the age of 50 [2], and the incidence rates for RC will increase by 124.2% for patients aged 20 to 34 years by 2030 [3].

The accurate staging of RC is essential because its treatment is highly stage-dependent. Currently, the clinical management of RC is based on the TNM classification, where the presence of regional lymph node metastases (N category) often distinguishes early-stage from locally advanced disease, which warrants neoadjuvant chemo/radiotherapy (NAT) [4]. In fact, the pathologic nodal status (pN) is one of the strongest prognostic factors in RC since patients with lymph node involvement are known to be at a higher risk of local recurrence and worse overall survival [5]. Therefore, the reliable preoperative prediction of nodal metastases is of great clinical importance, as it could inform treatment planning and improve patient stratification.

Currently, magnetic resonance imaging (MRI) plays a pivotal role in the preoperative evaluation of RC. High-resolution pelvic MRI (especially T2-weighted images) is the gold standard for assessing the primary tumor’s depth of invasion (T stage) and the status of the mesorectal fascia, which guides surgical decision-making [6,7]. However, accurately identifying metastatic lymph nodes in MRI remains a well-recognized challenge [8].

Conventional MRI criteria for nodal involvement—such as nodal size, border irregularity, or signal heterogeneity—are limited in sensitivity and specificity [9,10]. In fact, microscopic metastases can occur in normal-sized nodes, and enlarged nodes may be reactive rather than malignant, leading to substantial overlap in imaging appearances. The clinical implications of correctly classifying the lymph node staging in RC are significant, since the better preoperative prediction of the pN status could optimize treatment selection. For instance, pN+ patients may benefit from NAT and, conversely, pN− patients could avoid unnecessary overtreatment. The limitations in our current capacity to adequately predict the pN status has motivated ongoing research efforts in exploring new analytical approaches to augment the reliable non-invasive identification of pathological lymph nodes.

In the past decade, radiomics has emerged as an innovative strategy to extract a large number of quantitative features from medical images, converting them into high-dimensional minable data [11]. The central hypothesis of radiomics is that medical images contain latent information reflecting underlying tumor biology and heterogeneity, which can be decoded through computational analysis [12]. In the context of RC, MRI-based radiomics has shown promising results for improving risk stratification and guiding patient management. Several studies have demonstrated that quantitative MRI features can reflect important histopathological characteristics of rectal tumors. For example, radiomic analyses of pre-treatment MRI have been able to distinguish different T stages [13] or even predict pathologic complete response [14].

However, MRI-based radiomics accounts for several limitations that mainly derive from methodological variability, including differences in the MRI equipment used (e.g., 1.5 T vs. 3.0 T), technical acquisition parameters (e.g., echo time, repetition time, slice thickness), tumor segmentation process (e.g., manual, automatic with different software packages), or regions of interest (e.g., tumor alone, mesorectal compartment, lymph nodes alone for pN). In addition, data processing entails many potential sources of heterogeneity, including the selection of radiomics features, training/set partitions, use of cross-validation, or the set of machine learning (ML) methods available for model training. Further complicating matters, ongoing changes in the clinical management of RC introduce constraints to the longitudinal validity of published models.

**Figure 1:**
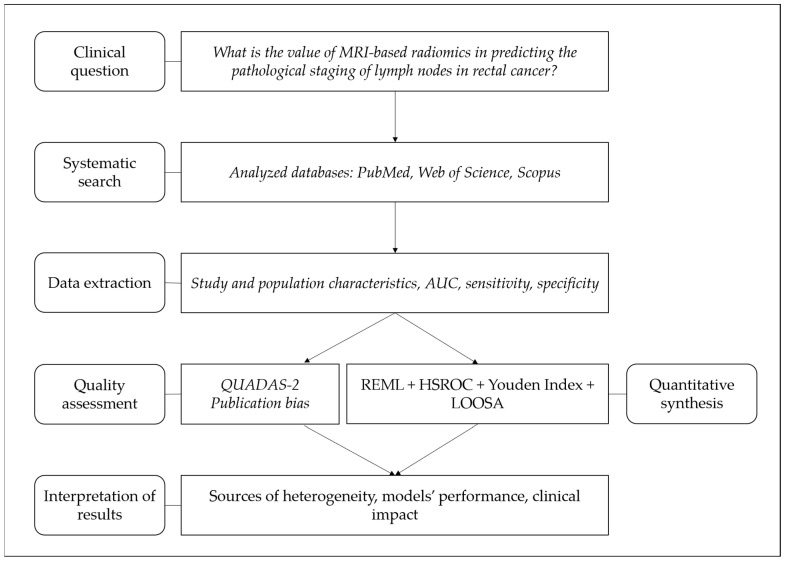
Flowchart of the steps and main aspects covered in this systematic review and meta-analysis.

Considering the promising results of MRI-based radiomics but also the aforementioned limitations, the periodical up-to-date synthesis of available evidence is necessary to monitor advancements in this field. The aim of this study was to conduct a systematic review with meta-analysis of observational studies in which MRI-based radiomics models were used to predict the pathological nodal status of RC patients. A summary of the steps and main points covered in this study is shown in Figure 1.

## 2. Materials and Methods

### 2.1. Review Design and Eligibility Criteria

The research question addressed in this systematic review and meta-analysis was based on the PICO strategy. The population was defined as adult patients with histologically proven RC in whom MRI-based radiomics models were used to predict pathological nodal status. The design and writing of this study followed the Preferred Reporting Items for Systematic Reviews and Meta-Analysis (PRISMA) guidelines [15]. The PRISMA checklist can be consulted in Supplementary File S1. The protocol study was registered in the PROSPERO database (ref: CRD42025630824).

The inclusion criteria comprised original research publications that applied radiomics models based on RC MRI before surgery to predict the pathological status of lymph nodes (pN). Exclusion criteria were studies applying radiomics based on imaging techniques other than MRI, studies that did not report performance metrics or sufficient information for their reliable estimation, and studies solely reporting outcome metrics for models combining radiomics with other variables (e.g., clinical or radiological). In addition, case reports, editorials, and other article formats different from original studies were excluded. The search was conducted from the earliest available records to 31 December 2024.

### 2.2. Information Sources and Search Strategy

Two authors (MGC and DLG) searched the PubMed, Web of Science, and Scopus databases. Different search equations were used, and a final consistent equation was constructed for each database (Supplementary Files S2–S4). All titles and abstracts of interest were screened, and those which did not meet the eligibility criteria were excluded. Next, the screened studies were read in full to assess whether they met all eligibility criteria. Discrepancies during the article selection process were solved by a third author (AJLRB). To increase the sensitivity of the search, cluster and snowballing searches were also performed, examining the studies referenced by and referencing the fully read articles, respectively. Regarding the snowballing search, the examined publications were identified from bespoke sections such as ‘Cited by’ in PubMed and the remaining databases.

### 2.3. Measured Variables

For each study, the main characteristics were collected, including the first author, year, country, sample size, training/test size, age and sex distribution, MRI magnetic field, NAT administration, and validation methods. The primary outcomes were the AUC, specificity, sensitivity, and their confidence intervals. Additionally, the diagnostic odds ratio (DOR) was calculated as explained below. For studies evaluating multiple ML methods or different regions of interest for segmentation, each one was included as a separated entry in the analyses.

### 2.4. Data Extraction

Two authors (MGC and DLG) independently extracted the data from the selected articles, and a third author (AJLRB) reviewed the data and solved any discrepancies. All data were annotated in a spreadsheet for ulterior analysis. When not directly available, true positives (TP), true negatives (TN), false positives (FP), and false negatives (FN) were manually calculated using histology as the gold standard. In one case in which the number of pN cases was not explicitly provided for the training/test groups [16], we assumed a constant distribution between groups.

### 2.5. Quality Assessment

The QUADAS-2 tool [17] was used to systematically assess potential risks of bias and applicability concerns. For each study, two authors (MGC and DLG) classified the risk of bias as low, unclear, or high in each of the four risk of bias items and in the three applicability concern items. In addition, an overall risk of bias estimation was provided by consensus. In case of discrepancy, a third author (AJLRB) was consulted. On the other hand, the publication bias was analyzed using funnel plots and Egger’s tests.

### 2.6. Statistical Analysis

For each diagnostic performance metric, meta-analyses were performed using random-effects models to account for between-study heterogeneity. First, a hierarchical summary receiver operating characteristic (HSROC) analysis was applied to jointly analyze sensitivity and specificity values across studies, using a bivariate random-effects approach. From the fitted HSROC model, we obtained an estimate of the area under the curve (AUC), as well as pooled values of sensitivity, specificity, and Youden’s J Index (Sensitivity + Specificity − 1). Forest plots were then generated for sensitivity and specificity.

To complement the HSROC analysis, we performed a univariate meta-analysis of the AUC values reported by each study, for which 95% confidence interval (95% CI) values were estimated based on standard errors (SEs) when missing in the original studies, as explained below. We proceeded analogously to perform a random-effects meta-analysis of the diagnostic odds ratio (DOR), according to the following formula:

(1) $$DOR=\frac{TP\;\cdot TN}{FP\;\cdot \;FN}$$

The restricted maximum likelihood estimator was used to estimate the between-study variance, and the Hartung–Knapp–Sidik–Jonkman [18] method was applied to adjust the confidence intervals of the pooled estimates. Of note is that the logarithmic transformation of the DOR (logDOR) was applied prior to meta-analysis to normalize the distribution, and results were back-transformed for interpretation. Forest plots were generated for the AUC, DOR, and logDOR. Although heterogeneity statistics were not displayed in the plots, they were computed for each model, including τ^2^ and I^2^, and were used to confirm the adequacy of random-effects modeling in all cases.

If a study did not provide 95% CI nor SE values for sensitivity, specificity, or the AUC, we estimated them using principles of binomial distribution, following the method described by Brown et al. [19]:

(2) $$Binomial\;distribution=\sqrt{\frac{p\;(1-p)}{n}}$$

where p is the estimated proportion (e.g., sensitivity or specificity), and n is the number of samples of interest. The values of p and n vary depending on the metric under consideration. For sensitivity and specificity, the formulae derived from (2) are the following:

(3) $${SE}_{Sensitivity}=\sqrt{\frac{Sensitivity\;(1-Sensitivity)}{N^{+}}}$$

(4) $${SE}_{Specificity}=\sqrt{\frac{Specificity\;(1-Specificity)}{N^{-}}}$$

where N^+^ and N^−^ correspond to the number of positive and negative pN values included in the population (i.e., test groups), respectively. For SE estimation in AUCs, the formula proposed by Hanley and McNeil [20] was applied, as in previous studies [21]:

(5) $${SE}_{AUC}=\sqrt{\frac{AUC\;\cdot \left(1-AUC\right)+(N^{+}-1)\;\cdot (Q_{1}-{AUC}^{2})+(N^{-}-1)\;\cdot (Q_{2}-{AUC}^{2})}{N^{+}\cdot \;N^{-}}}$$

(6) $$\mathrm{where}\;\;\;\;\;\;\;\;\;\;\;\;\;\;\;\;\;\;\;\;Q_{1}=\frac{AUC}{2-AUC}\mathrm{and}\;Q_{2}=\frac{2\;\cdot \;{AUC}^{2}}{1+AUC}$$

After calculating SE values, 95% confidence intervals (95% CIs) for each outcome metric were estimated according to the following formula:

(7) 95%CI=P ±1.96 ·SE

in which P represents the estimated value of the metric of interest (i.e., sensitivity, specificity, and AUC).

Additionally, leave-one-out sensitivity analyses were performed to assess the influence of each individual study on the pooled logDOR and AUC values. Finally, publication bias was assessed by visual inspection of funnel plots and Egger’s tests based on the calculated logDOR values of the included studies.

## 3. Results

### 3.1. Search Results and Main Characteristics of the Studies

**Figure 2:**
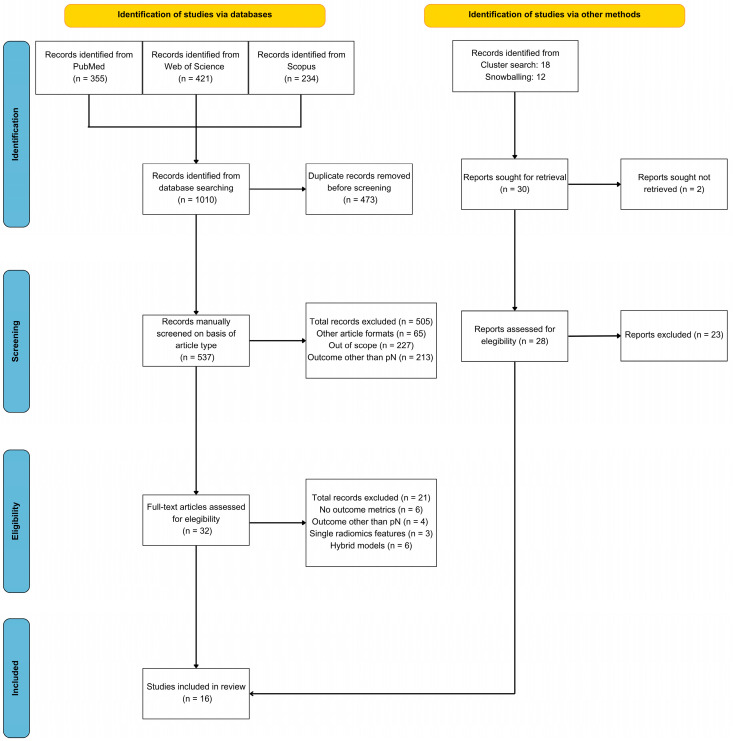
PRISMA flow diagram of the systematic review and meta-analysis.

The initial search in the three databases identified a total of 1010 articles. After duplicate removal (473 articles) and title/abstract screening, 32 articles were fully read. Following the inclusion and exclusion criteria, a total of 11 studies were finally included. Five additional articles were included following cluster and snowballing searches, leading to a total of 16 articles finally included in the meta-analysis. The PRISMA flow chart of this study can be consulted in Figure 2.

All the included studies [16,22,23,24,25,26,27,28,29,30,31,32,33,34,35,36] were performed in China with a retrospective design, except for one study [24], which was prospective. The total number of subjects included in this meta-analysis was 3157, ranging from 83 [22] to 391 [35] in each individual study. In most studies, the median age was close to 60 years old, except for one study [35] with a lower mean age of 53.67. Regarding sex distribution, all but two studies [23,30] included a lower number of women. All studies were performed using 1.5 or 3.0 T MRI machines, the latter being more frequent. Two studies included imaging studies performed using both 1.5 T and 3.0 T MRI scanners. Most studies employed at least T2-weighted images as the source for radiomics feature extraction, with nine studies including at least one additional sequence, including DWI, ADC, (non)contrast-enhanced T1 (CET1), and amide-proton transferase (APT)-weighted images. One study [25] used DWI sequences instead of T2 ones for segmentation and radiomics feature extraction. All studies except one [24] split the dataset into at least training and test sets. Three studies included an external validation cohort, while the remainder used an internal validation cohort with cross-validation. The main characteristics of each study are described in Table 1. Further information on each study can be consulted in Supplementary File S5.

### 3.2. Quality Assessment of the Included Studies

**Figure 3:**
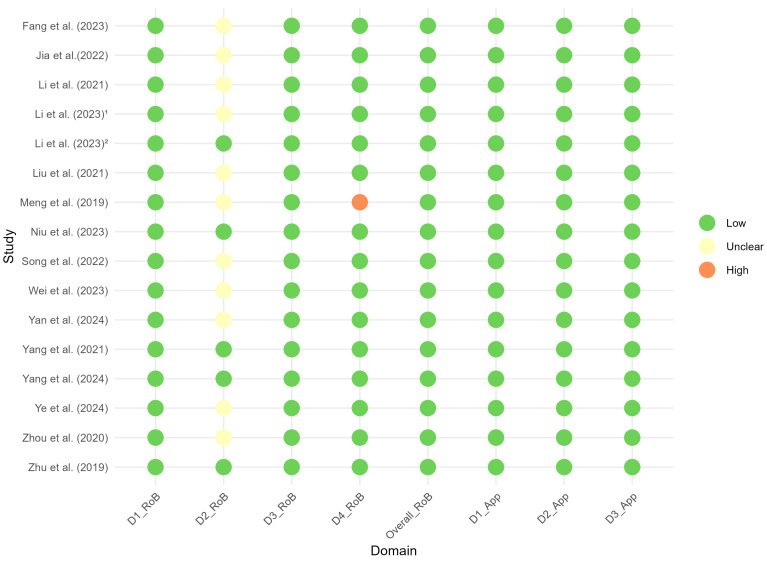
Traffic light plot of the QUADAS-2 assessment for the studies included in this systematic review and meta-analysis. Superscript numbers 1 and 2 in the rows related to Li et al. represent different references. Fang et al. (2023) [22], Jia et al. (2022) [23], Li et al. (2021) [24], Li et al. (2023) [25], Li et al. (2023) [26], Liu et al. (2021) [27], Meng et al. (2019) [28], Niu et al. (2023) [29], Song et al. (2022) [30], Wei et al. (2023) [16], Yan et al. (2024) [31], Yang et al. (2021) [32], Yang et al. (2024) [33], Ye et al. (2024) [34], Zhou et al. (2020) [35], Zhu et al. (2019) [36].

Figure 3 presents the results of the quality assessment performed with the QUADAS-2 tool. In general, the studies demonstrated a low risk of bias and few concerns about applicability in the evaluated domains. However, some uncertainty was observed in areas such as index test applicability and reference standard applicability. In particular, in 10 studies, the risk of bias in the index test domain was rated as “unclear” due to insufficient reporting on how the radiomics model was interpreted or whether blinding to the reference standard was ensured. Only one study [28] was judged to entail a potentially high risk of bias in one domain (flow and timing) because the dataset was split into training (discovery) and test sets based on a temporal criterion without sufficient justification. Further details on the assessment of risk of bias and applicability concerns domains can be found in Supplementary File S6.

### 3.3. Meta-Analysis of Sensitivity, Specificity, and Area Under the Curve

**Figure 4:**
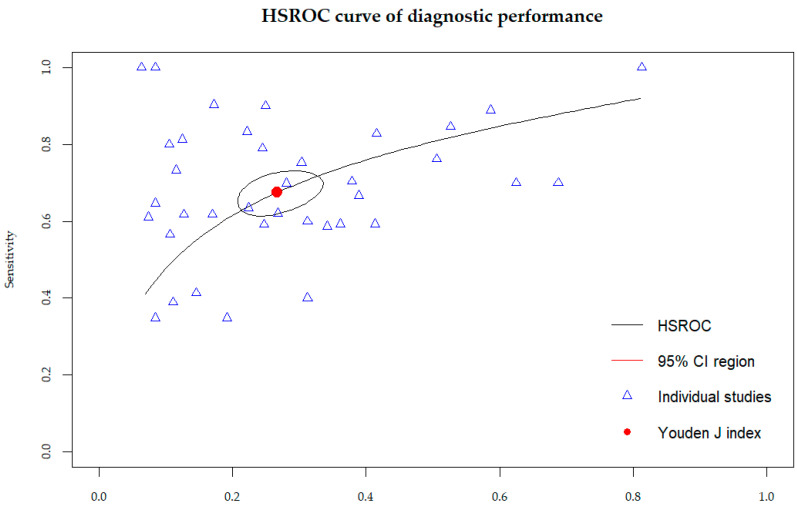
Hierarchical summary receiver operating characteristic (HSROC) curve of diagnostic performance of radiomics models analyzed in included studies.

**Figure 5:**
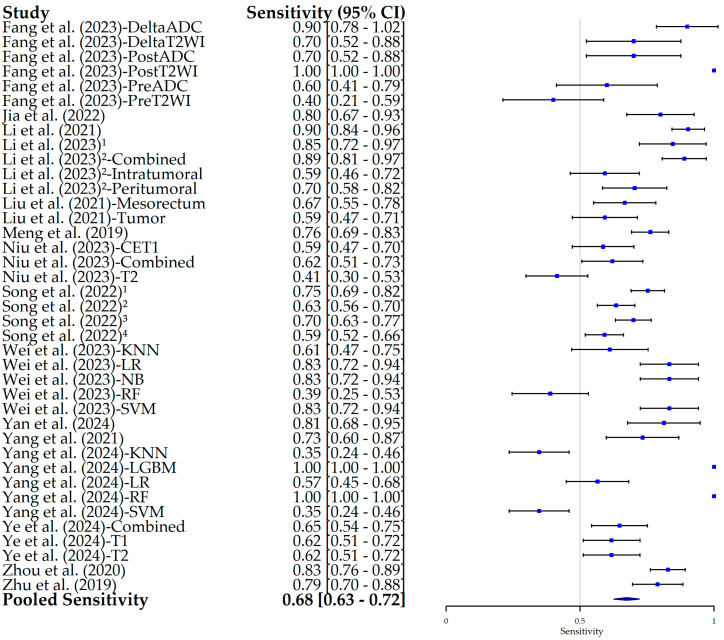
Forest plot of the pooled estimates of sensitivity and its 95% confidence interval (95% CI) for each individual study included in this meta-analysis. The squares correspond to the specificity values, and the lines to their 95% CIs. The diamond represents the pooled estimate value. Superscript numbers in the rows related to Song et al. (2022) [30] correspond to four different segmentation methods reported in the study, as follows: 1 along the border of lymph nodes; 2 an expanded border of 2–3 mm; 3 covering the border of lymph nodes; 4 circle regions within lymph nodes. Superscript numbers 1 and 2 in the rows related to Li et al. represent different references. Fang et al. (2023) [22], Jia et al. (2022) [23], Li et al. (2021) [24], Li et al. (2023) [25], Li et al. (2023) [26], Liu et al. (2021) [27], Meng et al. (2019) [28], Niu et al. (2023) [29], Song et al. (2022) [30], Wei et al. (2023) [16], Yan et al. (2024) [31], Yang et al. (2021) [32], Yang et al. (2024) [33], Ye et al. (2024) [34], Zhou et al. (2020) [35], Zhu et al. (2019) [36].

**Figure 6:**
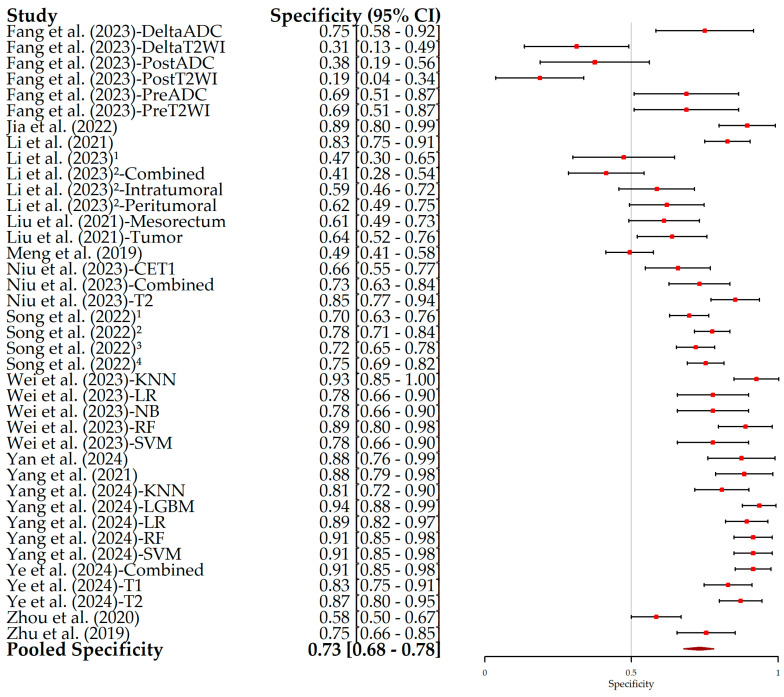
Forest plot of specificity and its 95% confidence interval (95% CI) for each individual study included in this meta-analysis. The squares correspond to the specificity values, and the lines to their 95% CIs. The diamond represents the pooled estimate value. Superscript numbers in the rows related to Song et al. (2022) [30] correspond to four different segmentation methods reported in the study, as follows: 1 along the border of lymph nodes; 2 an expanded border of 2–3 mm; 3 covering the border of lymph nodes; 4 circle regions within lymph nodes. Superscript numbers 1 and 2 in the rows related to Li et al. represent different references. Fang et al. (2023) [22], Jia et al. (2022) [23], Li et al. (2021) [24], Li et al. (2023) [25], Li et al. (2023) [26], Liu et al. (2021) [27], Meng et al. (2019) [28], Niu et al. (2023) [29], Song et al. (2022) [30], Wei et al. (2023) [16], Yan et al. (2024) [31], Yang et al. (2021) [32], Yang et al. (2024) [33], Ye et al. (2024) [34], Zhou et al. (2020) [35], Zhu et al. (2019) [36].

**Figure 7:**
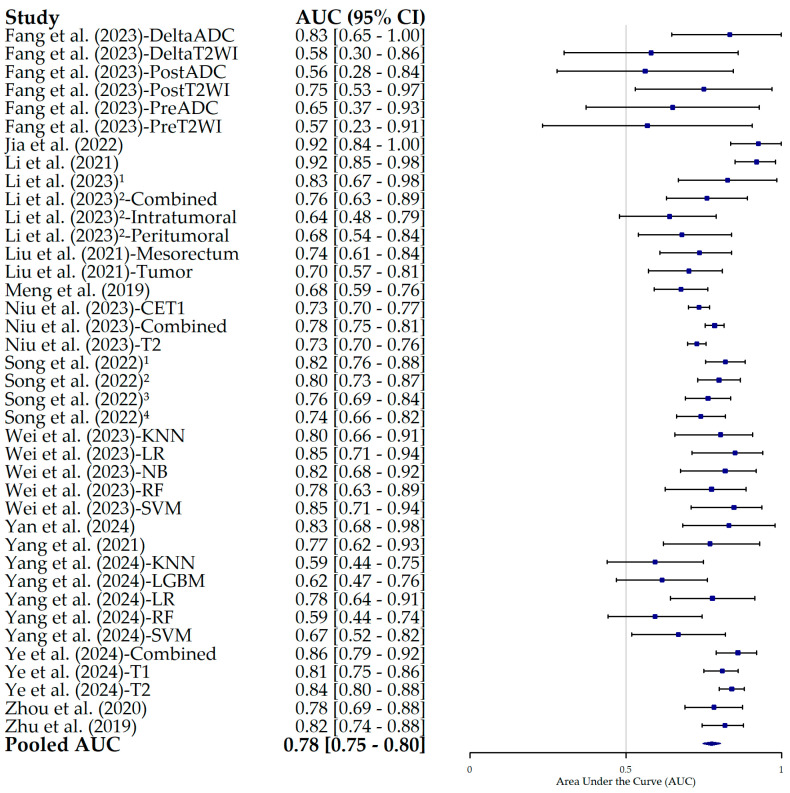
Forest plot of the pooled estimates of the area under the curve (AUC) and its 95% confidence interval (95% CI) for each individual study included in this meta-analysis. The squares correspond to the specificity values, and the lines to their 95% CIs. The diamond represents the pooled estimate value. Superscript numbers in the rows related to Song et al. (2022) [30] correspond to four different segmentation methods reported in the study, as follows: 1 along the border of lymph nodes; 2 an expanded border of 2–3 mm; 3 covering the border of lymph nodes; 4 circle regions within lymph nodes. Superscript numbers 1 and 2 in the rows related to Li et al. represent different references. Fang et al. (2023) [22], Jia et al. (2022) [23], Li et al. (2021) [24], Li et al. (2023) [25], Li et al. (2023) [26], Liu et al. (2021) [27], Meng et al. (2019) [28], Niu et al. (2023) [29], Song et al. (2022) [30], Wei et al. (2023) [16], Yan et al. (2024) [31], Yang et al. (2021) [32], Yang et al. (2024) [33], Ye et al. (2024) [34], Zhou et al. (2020) [35], Zhu et al. (2019) [36].

The results of the HSROC analysis (Figure 4) showed the moderate–high diagnostic performance of radiomics models in predicting the pN status in RC patients, with an AUC of 0.70 (95% CI, 0.65–0.75), a sensitivity of 0.68 (95% CI, 0.63–0.72) (Figure 5), and a specificity of 0.73 (95% CI, 0.68–0.78) (Figure 6). The complementary random-effects meta-analysis of the AUC showed a slightly higher pooled value of 0.78 (95% CI, 0.75–0.80), ranging from 0.56 in the model leveraging radiomics features from post-NAT ADC values in the study by Fang et al. (2022) [22] to 0.92 in the studies by Jia et al. (2022) [23] and Li et al. (2021) [24]. Figure 7 shows the corresponding forest plot.

**Figure 8:**
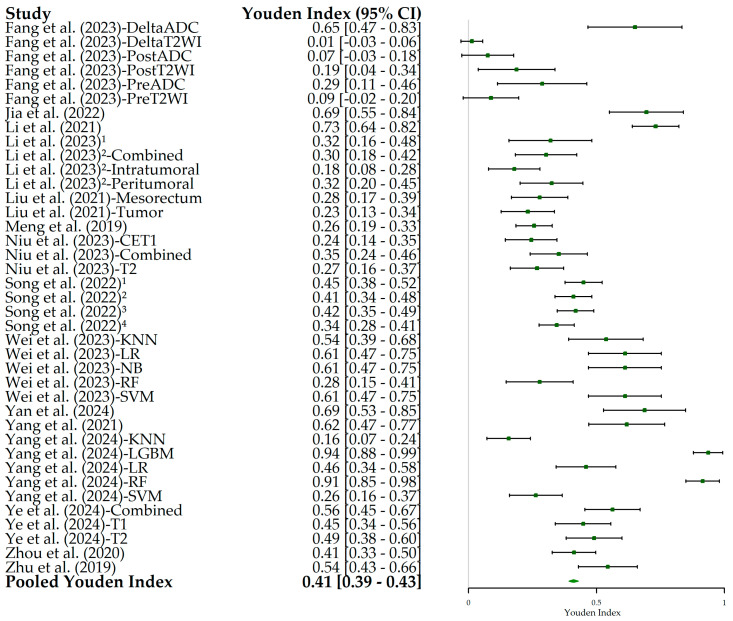
Forest plot of Youden’s index and its 95% confidence interval (95% CI) for each individual study included in this meta-analysis. The squares correspond to the Youden index values, and the lines to their 95% CIs. The diamond represents the pooled estimate value. The diamond represents the pooled estimate value. Superscript numbers in the rows related to Song et al. (2022) [30] correspond to four different segmentation methods reported in the study, as follows: 1 along the border of lymph nodes; 2 an expanded border of 2–3 mm; 3 covering the border of lymph nodes; 4 circle regions within lymph nodes. Superscript numbers 1 and 2 in the rows related to Li et al. represent different references. Fang et al. (2023) [22], Jia et al. (2022) [23], Li et al. (2021) [24], Li et al. (2023) [25], Li et al. (2023) [26], Liu et al. (2021) [27], Meng et al. (2019) [28], Niu et al. (2023) [29], Song et al. (2022) [30], Wei et al. (2023) [16], Yan et al. (2024) [31], Yang et al. (2021) [32], Yang et al. (2024) [33], Ye et al. (2024) [34], Zhou et al. (2020) [35], Zhu et al. (2019) [36].

The estimated Youden’s J index across studies (Figure 8) was 0.41, ranging from 0.01 in the DeltaT2WI radiomics model by Fang et al. (2023) [22] to 0.94 in the LGBM radiomics model by Yang et al. (2024) [33], indicating consistent diagnostic accuracy across studies.

### 3.4. Diagnostic Odds Ratio

**Figure 9:**
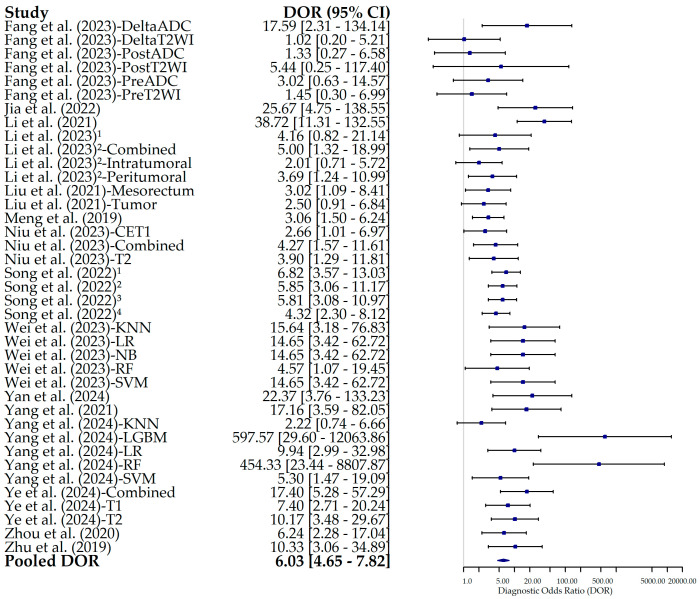
Forest plot of the diagnostic odds ratio (DOR) and its 95% confidence interval (95% CI) for each individual study included in this meta-analysis. The squares correspond to the DOR values, and the lines to their 95% CIs. The diamond represents the pooled estimate value. The diamond represents the pooled estimate value. Superscript numbers in the rows related to Song et al. (2022) [30] correspond to four different segmentation methods reported in the study, as follows: 1 along the border of lymph nodes; 2 an expanded border of 2–3 mm; 3 covering the border of lymph nodes; 4 circle regions within lymph nodes. Superscript numbers 1 and 2 in the rows related to Li et al. represent different references. Fang et al. (2023) [22], Jia et al. (2022) [23], Li et al. (2021) [24], Li et al. (2023) [25], Li et al. (2023) [26], Liu et al. (2021) [27], Meng et al. (2019) [28], Niu et al. (2023) [29], Song et al. (2022) [30], Wei et al. (2023) [16], Yan et al. (2024) [31], Yang et al. (2021) [32], Yang et al. (2024) [33], Ye et al. (2024) [34], Zhou et al. (2020) [35], Zhu et al. (2019) [36].

The DOR across studies ranged from 1.02 in the model using radiomics features from the differences between pre- and post-NAT T2WI in the study by Fang et al. (2023) [22] to 597.6 in the model using Light GBM in the study by Yang et al. (2024) [33]. The pooled DOR was 6.03 (95% CI: 4.65–7.82), indicating a moderate overall discriminatory capacity of radiomics models for presurgical nodal staging. Figure 9 presents the forest plot of the DOR across studies.

**Figure 10:**
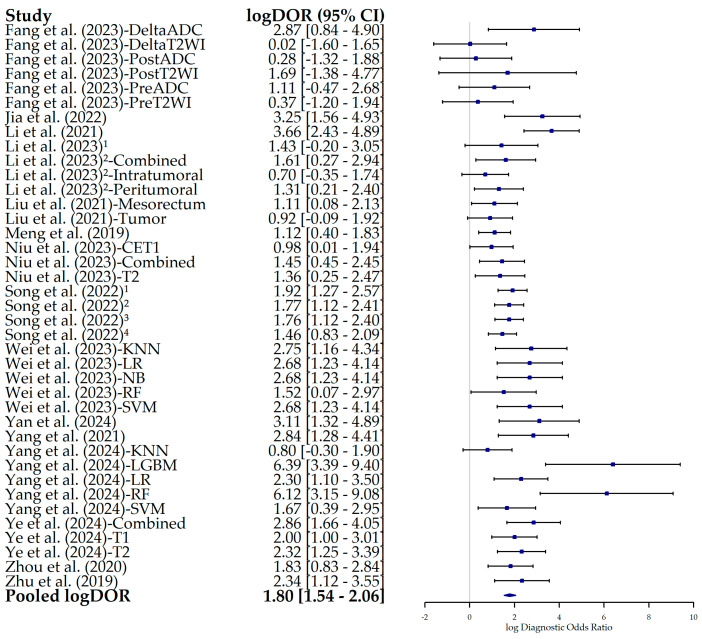
Forest plot of the logarithmic diagnostic odds ratio (logDOR) and its 95% confidence interval (95% CI) for each individual study included in this meta-analysis. The squares correspond to the logDOR values, and the lines to their 95% CIs. The diamond represents the pooled estimate value. The diamond represents the pooled estimate value. Superscript numbers in the rows related to Song et al. (2022) [30] correspond to four different segmentation methods reported in the study, as follows: 1 along the border of lymph nodes; 2 an expanded border of 2–3 mm; 3 covering the border of lymph nodes; 4 circle regions within lymph nodes. Superscript numbers 1 and 2 in the rows related to Li et al. represent different references. Fang et al. (2023) [22], Jia et al. (2022) [23], Li et al. (2021) [24], Li et al. (2023) [25], Li et al. (2023) [26], Liu et al. (2021) [27], Meng et al. (2019) [28], Niu et al. (2023) [29], Song et al. (2022) [30], Wei et al. (2023) [16], Yan et al. (2024) [31], Yang et al. (2021) [32], Yang et al. (2024) [33], Ye et al. (2024) [34], Zhou et al. (2020) [35], Zhu et al. (2019) [36].

The associated forest plot for logDOR showed a pooled estimate of 1.80 (95% CI, 1.54–2.06) and ranged from 0.02 in the DeltaT2WI model by Fang et al. (2023) [22] to 6.39 in the LGBM model by Yang et al. (2024) [33] (Figure 10).

### 3.5. Sensitivity Analyses and Publication Bias

**Figure 11:**
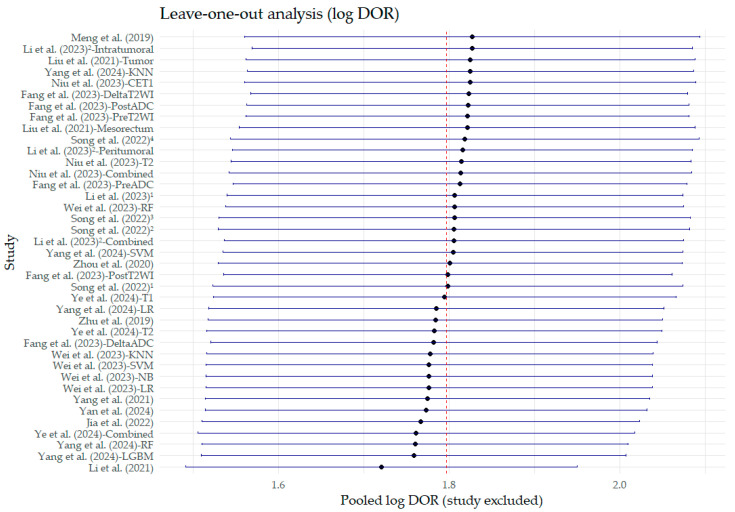
Leave-one-out sensitivity analysis of the pooled estimates of the logarithmic diagnostic odds ratio (log DOR). The diamond represents the pooled estimate value. Superscript numbers in the rows related to Song et al. (2022) [30] correspond to four different segmentation methods reported in the study, as follows: 1 along the border of lymph nodes; 2 an expanded border of 2–3 mm; 3 covering the border of lymph nodes; 4 circle regions within lymph nodes. Superscript numbers 1 and 2 in the rows related to Li et al. represent different references. Fang et al. (2023) [22], Jia et al. (2022) [23], Li et al. (2021) [24], Li et al. (2023) [25], Li et al. (2023) [26], Liu et al. (2021) [27], Meng et al. (2019) [28], Niu et al. (2023) [29], Song et al. (2022) [30], Wei et al. (2023) [16], Yan et al. (2024) [31], Yang et al. (2021) [32], Yang et al. (2024) [33], Ye et al. (2024) [34], Zhou et al. (2020) [35], Zhu et al. (2019) [36].

**Figure 12:**
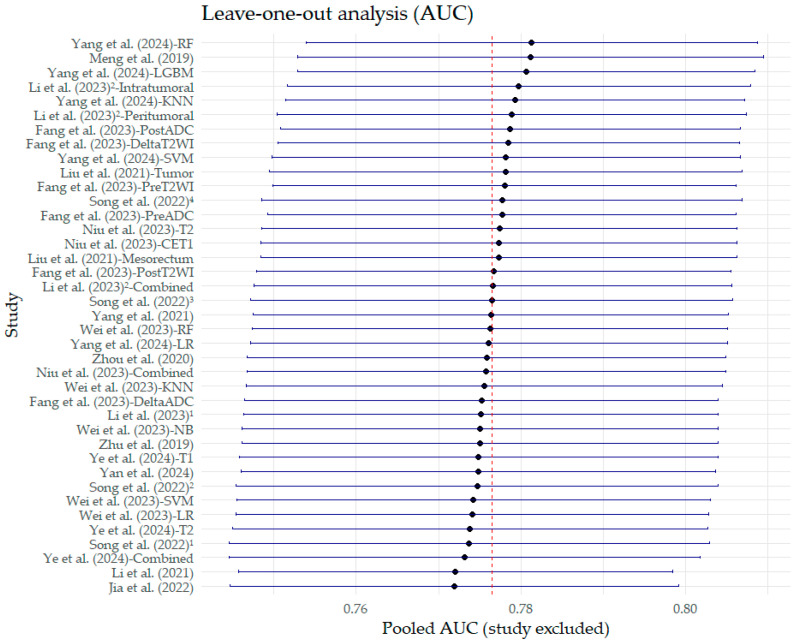
Leave-one-out sensitivity analysis on the pooled estimates of the area under the curve (AUC). The diamond represents the pooled estimate value. Superscript numbers in the rows related to Song et al. (2022) [30] correspond to four different segmentation methods reported in the study, as follows: 1 along the border of lymph nodes; 2 an expanded border of 2–3 mm; 3 covering the border of lymph nodes; 4 circle regions within lymph nodes. Superscript numbers 1 and 2 in the rows related to Li et al. represent different references. Fang et al. (2023) [22], Jia et al. (2022) [23], Li et al. (2021) [24], Li et al. (2023) [25], Li et al. (2023) [26], Liu et al. (2021) [27], Meng et al. (2019) [28], Niu et al. (2023) [29], Song et al. (2022) [30], Wei et al. (2023) [16], Yan et al. (2024) [31], Yang et al. (2021) [32], Yang et al. (2024) [33], Ye et al. (2024) [34], Zhou et al. (2020) [35], Zhu et al. (2019) [36].

Sensitivity analyses showed that the exclusion of the study by Li et al. (2021) [24] led to the most significant reduction in the logDOR, although the quantitative decrease was negligible (<0.1) (Figure 11). Similarly, in the AUC sensitivity analysis, none of the studies excluded significantly altered the pooled AUC values (Figure 12).

**Figure 13:**
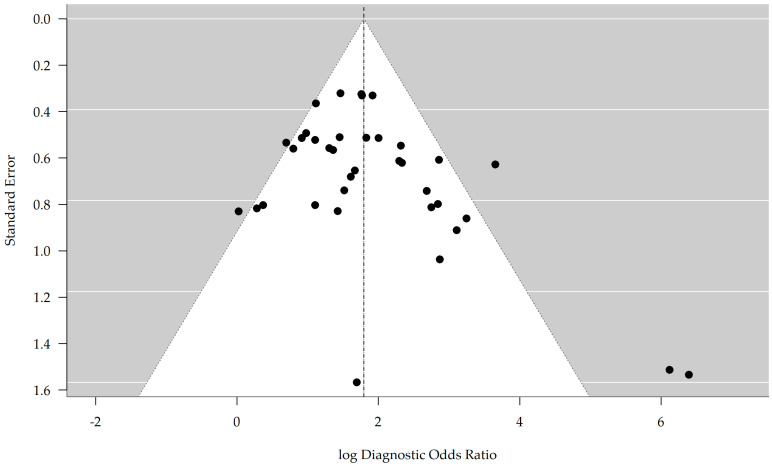
Funnel plot of the included studies based on the logarithmic (log) diagnostics odds ratio.

Egger’s test based on the logDOR estimates indicated a statistically significant association between the effect size and its standard error (t = 2.35; df = 37; *p* = 0.025), suggesting asymmetry in the distribution of studies. The estimated intercept as the standard error approached zero was 0.971 (95% CI, 0.275–1.666), indicating a potential small-study effect and the likelihood of an overestimation of the overall logDOR. These findings are suggestive of the presence of publication bias Figure 13.

## 4. Discussion

The increasing interest in developing reliable radiomics models to enhance the ability of radiologists to diagnose and predict oncological outcomes in RC patients has resulted in a significant number of publications in recent years (e.g., [37,38,39]). The aim of our meta-analysis was to synthesize the available evidence on MRI-based radiomics approaches to predict pN status in this context. Not only does the novelty and importance of this study lie in the up-to-date quantitative synthesis of state-of-the-art developments in this research field, but it also lies in particular advantages derived from the methodological approach followed: (1) we included studies specific to MRI-based radiomics (i.e., excluding other imaging technique sources such as computed tomography); (2) we focused on models exclusively exploiting radiomics information (i.e., models combining clinical and radiomics data were not considered in quantitative synthesis), thus providing specific information on the actual value of radiomics; (3) studies reporting outcomes from different ML models or radiomics-related analyses were incorporated into the meta-analysis as independent entries. Contrarily to previous meta-analyses (e.g., [40,41]), these methodological nuances translate into a more specific quantitative synthesis of the actual value of radiomics in predicting the pN status of patients with RC.

Our results show that radiomics holds promising potential as a complementary, quantitative analysis tool for pN staging in MRI. In particular, we identified a total of 16 studies with this particular focus, encompassing data from over 3000 patients. While the pooled sensitivity and specificity values of radiomics models were moderate, several of them achieved outstanding results with AUC values over 0.90. However, the lack of methodological uniformity and variability in outcome metrics represents a limitation that needs further consideration. Therefore, validation in larger multicentric cohorts and the standardization of radiomics methodologies will be essential before MRI-based radiomics can be adopted into routine practice.

In the studies included in this review, researchers extracted radiomics features not only from the primary tumor but also from lymph nodes or from the tumor’s surroundings (e.g., mesorectal fat) in MRI [24]. Interestingly, the results obtained consistently suggest that even features of the primary tumor alone can be informative of pN status. This approach is not novel and has been addressed earlier by histogram-feature (i.e., first-order statistics) analysis by authors such as Yang et al. (2019), who found that the T2-weighted signal intensity histogram of a tumor was significantly associated with regional nodal involvement [42]. However, we found a number of studies specifically targeting regional lymph nodes as a source to extract radiomics features. Li et al. (2023) [25] used lymph node-based segmentation radiomics as the basis for predictive model building, but they did not conduct node-by-node correlation. Instead, they considered pN+ status (at least one positive node in pathological report) as the ground truth for model building. A similar approach with slight modifications was followed by authors like Zhu et al. (2019) [36] and Yan et al. (2024) [31], the latter focusing on lateral lymph nodes.

An intrinsic problem of the latter approach lies in the fact that micrometastases in lymph nodes may lack MRI correlation on visual inspection by radiologists [43]. An alternative approach is to conduct a mapping of lymph nodes in a surgical specimen and correlate them with MRI findings. Obviously, this strategy limits clinical feasibility as it requires a complex radiological–pathological process. In spite of this, two studies were able to conduct such a difficult task. In the study by Song et al. (2022) [30], patients underwent surgery within 1 week of MRI examination. After the operation, the radiologist and pathologists jointly processed the resected specimens to locate the lymph nodes according to their relative position with other anatomical structures. Therefore, they were able to perform regression analyses of radiomics models based on a lymph node (instead of patient) category level. A similar yet less complicated approach was followed by Ye et al. (2024) [34]. In this case, after the (blinded) MRI classification of lymph nodes, they were individually matched with the descriptions (number and locations) provided in the pathology reports. Notably, only nodes >5 mm in short diameter were considered as pathological in the MRI report. Although this relatively heuristic approach may entail some biases (e.g., microscopic metastases in small-sized lymph nodes), it seems a simple yet useful strategy for MRI-based radiomics to predict pN status on a lymph node category-level basis.

Finally, authors such as Liu et al. (2021) [27] employed segmentations of the whole mesorectum to extract radiomics features. This represents a ‘clever’ approach, considering that the mesorectum mostly coincides with the surgical specimen in total mesorectal excision and thus contains most of the elements that are considered in local RC staging. In addition, this approach can be more generalizable because it avoids the need to specifically segment the tumor and lymph nodes—which are highly variable among patients. The main drawback of this segmentation strategy is the fact that such an extensive volume (i.e., the mesorectum) also includes a large number of non-relevant pixels (e.g., non-tumor areas, imaging artifacts), which may introduce significant noise [44]. Future studies could take advantage of this approach, provided that a sufficiently large and representative dataset is available.

On the other hand, our meta-analysis reflects the variability in the use of different MRI sequences to extract radiomics features. While T2-weighted images were a constant source of radiomics feature extraction across all studies—which seems logical considering its high resolution and fundamental role in the conventional radiological approach to RC staging—we found that (non)contrast-enhanced T1WI, T1-DCE, and DWI/ADC were also included in some multi-sequence-based radiomics models. For instance, four studies [27,28,33,35] extracted radiomics features from DWI images to complement T2-WI radiomics. Interestingly, one study [25] extracted features exclusively from DWI sequences, and their model showed moderate–high performance with an AUC value of 0.83 (95% CI, 0.67–0.98). On the other hand, two studies [22,23] used ADC-based features to construct radiomics models. An interesting approach was followed by Fang et al. (2023) [22], who constructed radiomics models based on pre-, post- and pre–post- (delta) NAT MRI radiomics features. Their top-performing model was deltaADC (AUC = 0.83), followed by post-T2WI (AUC = 0.75). Finally, contrast-enhanced MRI sequences were used by three studies [29,31,35], with AUC values ranging from 0.73 to 0.81, while one study [28] exploited T1-DCE sequences with modest results (AUC = 0.68). Thus, despite a number of potential confounders and lack of direct model comparison necessitating caution in the interpretation of results, DWI/ADC seem to offer more valuable information for lymph node status prediction than contrast-enhanced sequences. In fact, the highest AUC value (0.92) was found in the study by Jia et al. (2022) [23], who combined T2-WI and ADC values for radiomics extraction. Surprisingly, models incorporating information from more than three MRI sequences showed only moderate accuracy (e.g., AUC values of 0.68 in Meng et al., 2019 [28], or 0.78 in Zhou et al., 2020 [35]).

The results of our meta-analysis are consistent with those reported in previous published studies. For instance, Abbaspour et al. (2024) [40] recently conducted a meta-analysis on this same topic. Their results showed outstanding pooled AUC values of 0.81 in HSROC analysis. However, they applied significantly different criteria for study inclusion, and one paramount difference compared to our present study lies in the fact that they included different imaging techniques (e.g., CT and MRI). Moreover, they also included the results of the optimal radiomics model in each study, e.g., models combining clinical and radiomics data. These factors may have artificially overestimated the actual value of radiomics in this setting. Similarly, a previous meta-analysis by Bedrikovetski et al. (2021) [41] including both radiomics-based and deep learning-based approaches showed an almost identical AUC value (0.808) for radiomics studies and a promising AUC value of 0.917 for deep learning-based models, although only two original studies were considered in the latter group.

Despite the interest of such meta-analyses, the present study represents a further step toward the synthesis of state-of-the-art evidence-based results regarding the potential of radiomics as an isolated quantitative imaging analysis tool. In our opinion, the first step for introducing radiomics in clinical practice should be focused on homogenizing the methodological steps that may introduce significant variability in the development of models. Then, one could move toward integrating this approach with data from other sources (e.g., clinical, analytical, or from other omics) and establish appropriate comparisons with deep learning-based models. Notably, a minor exception to this was the inclusion of the study by Yang et al. (2024) [33], who combined radiomics with DL-based features. This study was retained because of the predominance of radiomics features and the explicability of the deep learning-based characteristics which are closer to radiomics than other deep learning approaches.

## 5. Limitations and Recommendations for Future Research

The present study has several limitations that need to be acknowledged. The first and most important one lies in the variability found in the methodological approaches followed across studies. Such heterogeneity may involve the different stages of the radiomics analysis workflow (e.g., segmentation process, segmentation targets, MRI sequences used), the use of ML approaches for predictive model selection, and other factors such as the characteristics of patients and tumors, the validation methodology, or the use of NAT. Although our meta-analytical approach allowed us to synthesize the results of studies with relatively consistent and homogeneous populations and approaches, it was impossible to control for all the factors that may act as a potential source of heterogeneity. However, our a priori decision to separate studies reporting outcomes from models obtained using different segmentation or ML approaches regardless of their precision yield allowed us to identify, for a given study, how some methodological differences altered the reported outcomes.

On the other hand, although most studies reported sufficient data to directly estimate AUC confidence intervals, one study required the approximation of N^+^ and N^−^ in the test subset (based on the distribution of the entire cohort). While sensitivity analyses indicated that this approximation had minimal influence on the overall results, it inherently introduced some degree of uncertainty. We encourage authors of future radiomics studies to report complete diagnostic contingency data to support reproducibility and meta-analytical integration. Notably, studies that only reported AUC values but not sufficient information for estimating AUC intervals reliably were excluded from our meta-analysis to avoid significant bias in pooled estimates.

In addition, although sensitivity and specificity are not directly affected by class imbalance in their definition, skewed N^+^/N^−^ distributions can influence model performance and generalizability, especially if not properly addressed during model development. While our meta-analysis incorporated class sizes in variance estimation, we acknowledge that some included studies may have been affected by imbalance-related biases. Future studies should report class distribution explicitly and describe any resampling or balancing strategies applied during training and validation.

The results of the publication bias analysis are also worth mentioning as a limitation and area of further research, as they suggest a positive publication bias; studies with worse results derived from smaller sample sizes were lacking. However, it should be noted that low sample sizes are a critical factor in studies based on ML approaches, potentially justifying their absence at the lower end of the funnel plot. Moreover, our methodological approach including the results of all models reported in the studies—rather than the top-performing ones—contributed to offer a wider and more representative panoramic view of radiomics performance in this clinical problem.

Importantly, some studies developed multiple radiomics models based on the same patient cohort, differing in segmentation strategy, MRI sequences, or ML algorithms. Each model was included as a separate entry in our meta-analysis following a model-based approach. Although this could introduce within-study clustering, we applied random-effects model analyses, which are more robust to such clustering, and performed leave-one-out sensitivity analyses, which confirmed the stability of the pooled estimates. Be that as it may, future meta-analyses with a larger number of studies should focus on subgroup and/or meta-regression analyses to identify how these factors may influence outcomes.

Finally, another limitation is that certain databases such as Embase and IEEE Xplore were not searched. Although substantial overlap exists between Embase, PubMed, and Scopus, we acknowledge that the inclusion of Embase could have increased retrieval sensitivity. Future systematic reviews and meta-analyses related to this research field may benefit from extending the search to these sources.

## 6. Conclusions

This updated meta-analysis supports the promising role of MRI-based radiomics as a complementary tool for the presurgical prediction of pathological lymph node status in RC staging. While the pooled sensitivity (0.68) and specificity (0.73) across studies were moderate, top-performing models showed very high precision yield (AUC > 0.85) and the pooled DOR (6.03) was high. Significant variability was found regarding anatomical regions of interest for segmentation, MRI sequences used as a source for radiomics feature extraction, and machine learning models applied. Moreover, cues of potential publication bias were identified, which could overestimate the actual precision of radiomics models and should be further explored in future studies.

Overall, our findings have significant implications for researchers, stakeholders and policymakers. For researchers, there is an urgent need for standardized radiomics workflows, robust external validation, and the transparent reporting of diagnostic performance. For developers and industry stakeholders, model explicability and reproducibility should be improved when designing tools for clinical use. Finally, for policymakers and guideline committees, our analysis suggests that MRI-based radiomics, although promising, is not yet ready for routine integration into clinical staging pathways for rectal cancer. Future efforts should focus on multi-institutional data sharing, harmonized pipelines, and the integration of radiomics into prospective trials to support clinical translation.

## Figures and Tables

**Table 1:** Main characteristics of the included studies.

Study (-Model)	Design	Country	Sample Size ^†^	Age *	Sex (%)	Validation Type	MRI Field	MRI Sequence(s)	N+ (Training)	N+ (Test)
Fang et al. (2023)-PreT2WI [22]	Retrospective	China	83	58.58–58.1	29	External validation cohort	1.5 T	T2, ADC	21/57 **	10/26
Fang et al. (2023)-PostT2WI [22]	Retrospective	China	83	58.58–58.1	29	External validation cohort	1.5 T	T2, ADC	21/57 **	10/26
Fang et al. (2023)-DeltaT2WI [22]	Retrospective	China	83	58.58–58.1	29	External validation cohort	1.5 T	T2, ADC	21/57 **	10/26
Fang et al. (2023)-PreADC [22]	Retrospective	China	83	58.58–58.1	29	External validation cohort	1.5 T	T2, ADC	21/57 **	10/26
Fang et al. (2023)-PostADC [22]	Retrospective	China	83	58.58–58.1	29	External validation cohort	1.5 T	T2, ADC	21/57 **	10/26
Fang et al. (2023)-DeltaADC [22]	Retrospective	China	83	58.58–58.1	29	External validation cohort	1.5 T	T2, ADC	21/57 **	10/26
Jia et al. (2022) [23]	Retrospective	China	126	59.60–65.33	51	External validation cohort	3.0 T	T2, ADC	32/87	20/39
Li et al. (2021) [24]	Prospective	China	91	59.31–61.19	40	5-fold CV (no test set)	3.0 T	T2	62/91	62/91
Li et al. (2023) ^1^ [25]	Retrospective	China	104	67.48 ± 9.96	34	Internal validation cohort (10-fold CV)	1.5 T	DWI	36/72	13/32
Li et al. (2023) ^2^-Intratumoral [26]	Retrospective	China	346	61.86 (26–88)	29	External validation cohort	1.5 and 3.0 T	T2	66/134	27/56
Li et al. (2023) ^2^-Peritumoral [26]	Retrospective	China	346	61.86 (26–88)	29	External validation cohort	1.5 and 3.0 T	T2	66/134	27/56
Li et al. (2023) ^2^-Combined [26]	Retrospective	China	346	61.86 (26–88)	29	External validation cohort	1.5 and 3.0 T	T2	66/134	27/56
Liu et al. (2021)-Tumor [27]	Retrospective	China	186	59.52 ± 11.44	32	Internal validation cohort	3.0 T	T2, DWI	54/123	27/63
Liu et al. (2021)-Mesorectum [27]	Retrospective	China	186	59.52 ± 11.44	32	Internal validation cohort	3.0 T	T2, DWI	54/123	27/63
Meng et al. (2019) [28]	Retrospective	China	345	59.48–61.10	39	Internal validation cohort (10-fold CV)	1.5 T	T1, T2, DWI, T1-DCE	62/190	63/146
Niu et al. (2023)-CET1 [29]	Retrospective	China	234	60.8 ± 9.7	40	Internal validation cohort (5-fold CV)	3.0 T	CET1	69/164	29/70
Niu et al. (2023)-T2 [29]	Retrospective	China	234	60.8 ± 9.7	40	Internal validation cohort (5-fold CV)	3.0 T	T2	69/164	29/70
Niu et al. (2023)-Combined [29]	Retrospective	China	234	60.8 ± 9.7	40	Internal validation cohort (5-fold CV)	3.0 T	CET1, T2	69/164	29/70
Song et al. (2022) ^1^ [30]	Retrospective	China	166	61.96 ± 11.03	63	Internal validation cohort (5-fold CV)	3.0 T	T2	215/422	93/182
Song et al. (2022) ^2^ [30]	Retrospective	China	166	61.96 ± 11.03	63	Internal validation cohort (5-fold CV)	3.0 T	T2	215/422	93/182
Song et al. (2022) ^3^ [30]	Retrospective	China	166	61.96 ± 11.03	63	Internal validation cohort (5-fold CV)	3.0 T	T2	215/422	93/182
Song et al. (2022) ^4^ [30]	Retrospective	China	166	61.96 ± 11.03	63	Internal validation cohort (5-fold CV)	3.0 T	T2	215/422	93/182
Wei et al. (2023)-NB [16]	Retrospective	China	125	61.40 ± 11.59	28	Internal validation cohort (5-fold CV)	3.0 T	T2, APT	23/56	18/45 ^
Wei et al. (2023)-KNN [16]	Retrospective	China	125	61.40 ± 11.59	28	Internal validation cohort (5-fold CV)	3.0 T	T2, APT	23/56	18/45 ^
Wei et al. (2023)-SVM [16]	Retrospective	China	125	61.40 ± 11.59	28	Internal validation cohort (5-fold CV)	3.0 T	T2, APT	23/56	18/45 ^
Wei et al. (2023)-RF [16]	Retrospective	China	125	61.40 ± 11.59	28	Internal validation cohort (5-fold CV)	3.0 T	T2, APT	23/56	18/45 ^
Wei et al. (2023)-LR [16]	Retrospective	China	125	61.40 ± 11.59	28	Internal validation cohort (5-fold CV)	3.0 T	T2, APT	23/56	18/45 ^
Yan et al. (2024) [31]	Retrospective	China	106	60.37 ± 12.17	34	Internal validation cohort (5-fold CV)	NS	T2	36/74	16/32
Yang et al. (2021) [32]	Retrospective	China	139	64 (34–86)	35	Internal validation cohort (10-fold CV)	3.0 T	T2	40/98	15/41
Yang et al. (2024)-LR [33]	Retrospective	China	356	61.61 ± 12.76	38	Internal validation cohort (5-fold CV)	1.5 and 3.0 T	T2, DWI	98/286	23/70
Yang et al. (2024)-LGBM [33]	Retrospective	China	356	61.61 ± 12.76	38	Internal validation cohort (5-fold CV)	1.5 and 3.0 T	T2, DWI	98/286	23/70
Yang et al. (2024)-KNN [33]	Retrospective	China	356	61.61 ± 12.76	38	Internal validation cohort (5-fold CV)	1.5 and 3.0 T	T2, DWI	98/286	23/70
Yang et al. (2024)-SVM [33]	Retrospective	China	356	61.61 ± 12.76	38	Internal validation cohort (5-fold CV)	1.5 and 3.0 T	T2, DWI	98/286	23/70
Yang et al. (2024)-RF [33]	Retrospective	China	356	61.61 ± 12.76	38	Internal validation cohort (5-fold CV)	1.5 and 3.0 T	T2, DWI	98/286	23/70
Ye et al. (2024)-T1 [34]	Retrospective	China	144	59 ± 10	33	Internal validation cohort	3.0 T	CET1	78/189	34/81
Ye et al. (2024)-T2 [34]	Retrospective	China	144	59 ± 10	33	Internal validation cohort	3.0 T	T2	78/189	34/81
Ye et al. (2024)-Combined [34]	Retrospective	China	144	59 ± 10	33	Internal validation cohort	3.0 T	CET1, T2	78/189	34/81
Zhou et al. (2020) [35]	Retrospective	China	391	53.67 ± 12.20	29	Internal validation cohort (10-fold CV)	1.5 T	T1, T2, DWI, CET1	58/261	29/130
Zhu et al. (2019) [36]	Retrospective	China	215	55.59–58.56	39	Internal validation cohort (5-fold CV)	3.0 T	T2	34/143	19/72

Superscript numbers in the rows related to Song et al. (2022) [30] correspond to four different segmentation methods reported in the study, as follows: ^1^ along the border of lymph nodes; ^2^ an expanded border of 2–3 mm; ^3^ covering the border of lymph nodes; ^4^ circle regions within lymph nodes. ^†^ Mismatch between the total sample size and the sum of training and test sample sizes owes to missing data or differences between validation/test sets or category-level (subjects vs. lymph nodes) descriptions, as reported in the original studies. * Age is expressed as the mean ± standard deviation or median (range) when provided for the whole sample or as mean–mean in the training and test groups, respectively, when not provided for the entire sample. ** The synthetic minority oversampling technique (SMOTE) was applied to balance N categories exclusively to the training test. ^ The N+ number was not specified for the test set; thus continuous distribution based on the reported total number of N+ in the sample was assumed. ADC, apparent diffusion coefficient. APT, amide-proton transferase-weighted images. CET1, contrast-enhanced T1-weighted image. CV, cross-validation. DCE, dynamic contrast enhancement. DWI, diffusion-weighted image. KNN, k-Nearest Neighbors. LGBM, light gradient boosting machine. LR, linear regression. NB, naïve Bays. RF, random forest. SVM, support vector machine.

## Data Availability

The data used in this manuscript are available in the Supplementary Files. Specific analysis data are available upon reasonable request to the corresponding author.

## Supplementary Materials

The following supporting information can be downloaded at https://www.mdpi.com/article/10.3390/bioengineering12070786/s1, Supplementary File S1: PRISMA checklist; Supplementary File S2: PubMed search strategy; Supplementary File S3: Scopus search strategy; Supplementary File S4: Web of Science search strategy; Supplementary File S5: Further information about the included studies; Supplementary File S6: Assessment of QUADAS-2 items of the included studies.

## List of Abbreviations

**Abbreviation**	**Full Term**
ADC	Apparent Diffusion Coefficient
APT	Amide Proton Transfer-Weighted Imaging
AUC	Area Under the Curve
CET1	Contrast-Enhanced T1-Weighted Imaging
(95%) CI	(95%) Confidence Interval
CV	Cross-Validation
DL	Deep Learning
(log)DOR	(Logarithmic) Diagnostic Odds Ratio
DWI	Diffusion-Weighted Imaging
FN	False Negatives
FP	False Positives
HSROC	Hierarchical Summary Receiver Operating Characteristic
KNN	k-Nearest Neighbor
LGBM	Light Gradient Boosting Machine
LR	Logistic Regression
ML	Machine Learning
MRI	Magnetic Resonance Imaging
NB	Naïve Bayes
NAT	Neoadjuvant Therapy
pN	Pathological Nodal Status
PRISMA	Preferred Reporting Items for Systematic Reviews and Meta-Analyses
QUADAS-2	Quality Assessment of Diagnostic Accuracy Studies 2
RC	Rectal Cancer
RF	Random Forest
ROI	Region of Interest
SE	Standard Error
SVM	Support Vector Machine
T1-DCE	T1 Dynamic Contrast-Enhanced
TN	True Negatives
TNM	Tumor–Node–Metastasis (classification)
TP	True Positives
