# Supplementary material for: The Value of MRI-Based Radiomics in Predicting the Pathological Nodal Status of Rectal Cancer: A Systematic Review and Meta-Analysis
Source: Bioengineering (Basel). 2025 Jul 21;12(7):786. doi: 10.3390/bioengineering12070786 (PMC12292859; doi:10.3390/bioengineering12070786)
Supplement: Supplementary file 1 [file bioengineering-12-00786-s001.zip › Supplementary File 5.pdf]

Table 2. Characteristics of the included studies (continuation from Table 1).

Study	Tumor charact.	NAT	Number of radiologists segmenting	MRI timing	ROI	Imaging normalization	N. extracted features	Radiomic features extraction	Feature stability test	Feature norm.	Feature selection (reduction of dimensionality)	ML method	N. of radiomic features in signature
Fang et al. (2023)	LARC	No	2 radiologists	Baseline (pre-NAT) + post-NAT (<2 months before surgery)	Tumor region	NS	1409 (x6)	PyRadiomics	Yes (20 patients (ICC >0.75)	Yes (Z-normalization)	Yes (outliers removal + SMOTE + Z-normalization, and RFE-LR)	SVM	2 features/model
Jia et al. (2022)	NS	No	2 radiologists	10 days before surgery	Tumor region	NS	828 (x2)	Analysis Kit, Kinectis Version 2.1, GE Healthcare)	Yes (20 cases, ICC >0.75)	NS	Yes (multifactor linear weighting)	No	3 features
Li et al. (2021)	NS	No	1 (+ supervised)	<2 weeks before surgery	Lymph nodes	Yes (resampling and Z-score normalization)	1409	PyRadiomics.	NS	NS	Yes (correlation, IMIA)	Not applied	10 features
Li et al. (2023)[1]	NS	No	1	NS (preoperative)	Lymph nodes	No	842	NS	Yes (30 cases, 2 radiologist)	NS	ICC (>0.7) + LASSO	Not applied	8
Li et al. (2023)[2]	NS	No	1 (+ supervised)	<2 weeks before surgery	Tumoral and peritumoral regions	Yes	1409 (x2)	PyRadiomics (embeeded in 3D Slicer module)	Yes (30 cases, ICC >0.75, 2 radiologists)	NS	LASSO	Not applied	Intratumoral: 4 features; Peritumoral: 5 features; Combined: 10 features
Liu et al. (2021)	NS	NS	1	<2 weeks before surgery	Tumor on T2 and DWI, and mesorectum	NS	1653 (x3)	PyRadiomics.	Yes (20 cases, 2 radiologists)	Yes (min-max scaling)	Spearman + LASSO	SVM	TR: 11 features; TMR: 13 features
Meng et al. (2019)	NS	No	2 radiologists	<2 weeks before surgery	Tumor region	Yes	2534/patient	Matlab + Open Access program**	Yes (ICC >0.75)	Yes (Z-normalization)	Yes (Pearson correlation + uni/multivariate ranking methods)	Yes (RF, SVM) + LASSO	10 features
Niu et al. (2023)	NS	No	2 radiologists	NS	Tumor region	Yes	1130 (x2)	PyRadiomics (embeeded in 3D Slicer module)	Yes (50 cases, ICC >0.80)	Yes (min-max scaling or Z-normalization)	Yes (recursive feature elimination)	Yes (RF, Gaussian, Adaboost, KNN, MLP)	CET1: 11 features<br>T2: 6 features
Song et al. (2022)	NS	No	1	1 week before surgery	Mesorectal lymph nodes (4 methods)	NS	1409	In-house Python software (NS)	Yes (45 cases, 2 radiologists)	NS	ICC (>0.75)	Not applied	42, 62, 65 and 35 (segmentation methods 1-4, respectively)

											Variance threshold + Select K best + LASSO		
Wei et al. (2023)	NS	No	2 radiologists	<2 weeks before surgery	Tumor region	Yes	1920 features/patient	PyRadiomics	Yes (ICC >0.75)	Yes (Z-normalization)	Yes (outliers replacement + Spearman + Multivariate LR	Yes (NB, RF, KNN, SVM, LR)	7 features
Yan et al. (2024)	Rectal cancer with TME+lateral lymh node resection	No	1 radiologist	<2 weeks before surgery	Visible lateral lymph nodes	NS	560 features/lymph node	PyRadiomics	NS	NS	Yes (LASSO + backward stepwise regression method)	No	4 features
Yang et al. (2021)	NS	No	2 radiologists	<1 week before surgery	Rectal tumor	Yes (Z-score)	396	AK software (Artifical Intelligence Kit V 3.0.0. R, GE Healthcare)	Yes (30 cases, ICC >0.75)	NS	Yes (maximum correlation-minimum redundancy + LASSO)	No	9 features
Yang et al. (2024)	NS	No	1 (+ supervisor)	<2 weeks before surgery	Rectal tumor	Yes	1834 features/sequence (x2) + 180 DL features/sequence (x2)	PyRadiomics.	Yes (40 cases, 1 radiologist)	Yes (Z-normalization)	ICC (>0.75) + Mann-Whitney + Spearkman + LASSO	KNN + SVM + Light GBM + LR + RF	22 radiomics feature + 22 DL feature
Ye et al. 2024	NS	No	2	<2 weeks before surgery	Regional lymph nodes 5-10 mm	Yes	2000 features in total	PyRadiomics.	Yes (all cases)	Yes (Z-normalization)	LASSO<br><br>Dice coefficient (>0.75)	Not applied	T1WI: 13 features; T2WI: 10 features; T1+T2WI: 18
Zhou et al. 2020	LARC [T3-T4 + or N+]	Yes	2	After NAT	Tumor	No	264 for each sequence (x4)	In-house MATLAB (MathWorks, Inc., Natick, MA, USA) software	No	Yes (Z-normalization)	Wilcoxon test + Spearman + LASSO	Not applied	13
Zhu et al. (2019)	LARC	Yes	1 radiologist	Before and after NAT	Lymph nodes	Yes (Z-normalization)	412/lymph node	In-house MATLAB (MathWorks, Inc., Natick, MA, USA) software	Yes (50 cases, ICC -values obtained over 0.80)	NS	Voxels with intensities outside μ±3δ were excluded from feature extraction + t test + LASSO	No	7 features

*IMIA: iterative multi-objective immune algorithm. CET1, contrast-enhanced T1-weighted image. ICC, intraclass correlation coefficient. KNN, K-Nearest Neighbors. LARC, locally advanced rectal cancer. LASSO, Least Absolute Shrinkage and Selection Operator. NAT, neoadjuvant therapy. NS, not specified. RFE-LR, recursive feature elimination with logistic regression. RF, random forest.

**The URL provided by the authors is https://github.com/mvallieres/radiomics/.
