# Supplementary material for: The Value of MRI-Based Radiomics in Predicting the Pathological Nodal Status of Rectal Cancer: A Systematic Review and Meta-Analysis
Source: Bioengineering (Basel). 2025 Jul 21;12(7):786. doi: 10.3390/bioengineering12070786 (PMC12292859; doi:10.3390/bioengineering12070786)
Supplement: Supplementary file 1 [file bioengineering-12-00786-s001.zip › Supplementary File 2.pdf]

Supplementary Material 1
Research strategy for Pubmed

("radiomic*"[All Fields] OR ("textural"[All Fields] OR "texturally"[All Fields] OR "texture"[All Fields] OR "texture s"[All Fields] OR "textured"[All Fields] OR "textures"[All Fields] OR "texturing"[All Fields] OR "texturization"[All Fields] OR "texturize"[All Fields] OR "texturized"[All Fields] OR "texturizing"[All Fields])) AND ("magnetic resonance imaging"[MeSH Terms] OR ("magnetic"[All Fields] AND "resonance"[All Fields] AND "imaging"[All Fields]) OR "magnetic resonance imaging"[All Fields] OR ("magnetic resonance imaging"[MeSH Terms] OR ("magnetic"[All Fields] AND "resonance"[All Fields] AND "imaging"[All Fields]) OR "magnetic resonance imaging"[All Fields] OR "mri"[All Fields])) AND ("rect*"[All Fields] AND ("cancer s"[All Fields] OR "cancerated"[All Fields] OR "canceration"[All Fields] OR "cancerization"[All Fields] OR "cancerized"[All Fields] OR "cancerous"[All Fields] OR "neoplasms"[MeSH Terms] OR "neoplasms"[All Fields] OR "cancer"[All Fields] OR "cancers"[All Fields] OR ("cysts"[MeSH Terms] OR "cysts"[All Fields] OR "cyst"[All Fields] OR "neurofibroma"[MeSH Terms] OR "neurofibroma"[All Fields] OR "neurofibromas"[All Fields] OR "tumor s"[All Fields] OR "tumoral"[All Fields] OR "tumorous"[All Fields] OR "tumour"[All Fields] OR "neoplasms"[MeSH Terms] OR "neoplasms"[All Fields] OR "tumor"[All Fields] OR "tumour s"[All Fields] OR "tumoural"[All Fields] OR "tumourous"[All Fields] OR "tumours"[All Fields] OR "tumors"[All Fields]) OR ("neoplasm s"[All Fields] OR "neoplasms"[MeSH Terms] OR "neoplasms"[All Fields] OR "neoplasm"[All Fields])))
