# Supplementary material for: The Value of MRI-Based Radiomics in Predicting the Pathological Nodal Status of Rectal Cancer: A Systematic Review and Meta-Analysis
Source: Bioengineering (Basel). 2025 Jul 21;12(7):786. doi: 10.3390/bioengineering12070786 (PMC12292859; doi:10.3390/bioengineering12070786)
Supplement: Supplementary file 1 [file bioengineering-12-00786-s001.zip › Supplementary File 3.pdf]

Supplementary File 3

Research strategy for SCOPUS

(TITLE-ABS-KEY(radiomic*) OR TITLE-ABS-KEY(textural) OR TITLE-ABS-KEY(texturally) OR TITLE-ABS-KEY(texture) OR TITLE-ABS-KEY(textured) OR TITLE-ABS-KEY(textures) OR TITLE-ABS-KEY(texturing) OR TITLE-ABS-KEY(texturization) OR TITLE-ABS-KEY(texturize) OR TITLE-ABS-KEY(texturized) OR TITLE-ABS-KEY(texturizing))
AND
(TITLE-ABS-KEY("magnetic resonance imaging") OR TITLE-ABS-KEY(mri))
AND
(TITLE-ABS-KEY(rect*) AND (TITLE-ABS-KEY(cancers) OR TITLE-ABS-KEY(cancer) OR TITLE-ABS-KEY(cancerous) OR TITLE-ABS-KEY(neoplasms) OR TITLE-ABS-KEY(tumor) OR TITLE-ABS-KEY(tumour) OR TITLE-ABS-KEY(tumoral) OR TITLE-ABS-KEY(tumorous) OR TITLE-ABS-KEY(cysts) OR TITLE-ABS-KEY(cyst) OR TITLE-ABS-KEY(neurofibroma) OR TITLE-ABS-KEY(neurofibromas)))
