# Supplementary material for: The Value of MRI-Based Radiomics in Predicting the Pathological Nodal Status of Rectal Cancer: A Systematic Review and Meta-Analysis
Source: Bioengineering (Basel). 2025 Jul 21;12(7):786. doi: 10.3390/bioengineering12070786 (PMC12292859; doi:10.3390/bioengineering12070786)
Supplement: Supplementary file 1 [file bioengineering-12-00786-s001.zip › Supplementary File 4.pdf]

Supplementary File 2

Research strategy for Web of Science

TS=(radiomic* OR textural OR texturally OR texture OR textured OR textures OR texturing OR texturization OR texturize OR texturized OR texturizing)
AND
TS=("magnetic resonance imaging" OR mri)
AND
TS=(rect* AND (cancers OR cancer OR cancerous OR neoplasms OR tumor OR tumour OR tumoral OR tumorous OR cysts OR cyst OR neurofibroma OR neurofibromas))
